# caCORRECT2: Improving the accuracy and reliability of microarray data in the presence of artifacts

**DOI:** 10.1186/1471-2105-12-383

**Published:** 2011-09-29

**Authors:** Richard A Moffitt, Qiqin Yin-Goen, Todd H Stokes, R Mitchell Parry, James H Torrance, John H Phan, Andrew N Young, May D Wang

**Affiliations:** 1 The Wallace H. Coulter Department of Biomedical Engineering, Georgia Institute of Technology and Emory University, 313 Ferst Drive, Atlanta, GA, 30332, USA; 2 Department of Pathology and Laboratory Medicine, Emory University School of Medicine, Grady Health System, Grady Memorial Hospital, Atlanta, GA 30303, USA; 3 Department of Electrical and Computer Engineering, Georgia Institute of Technology, Atlanta, GA 30332, USA; 4 Winship Cancer Institute, Emory University, Atlanta, GA 30322, USA

## Abstract

**Background:**

In previous work, we reported the development of caCORRECT, a novel microarray quality control system built to identify and correct spatial artifacts commonly found on Affymetrix arrays. We have made recent improvements to caCORRECT, including the development of a model-based data-replacement strategy and integration with typical microarray workflows via caCORRECT's web portal and caBIG grid services. In this report, we demonstrate that caCORRECT improves the reproducibility and reliability of experimental results across several common Affymetrix microarray platforms. caCORRECT represents an advance over state-of-art quality control methods such as Harshlighting, and acts to improve gene expression calculation techniques such as PLIER, RMA and MAS5.0, because it incorporates spatial information into outlier detection as well as outlier information into probe normalization. The ability of caCORRECT to recover accurate gene expressions from low quality probe intensity data is assessed using a combination of real and synthetic artifacts with PCR follow-up confirmation and the affycomp spike in data. The caCORRECT tool can be accessed at the website: http://cacorrect.bme.gatech.edu.

**Results:**

We demonstrate that (1) caCORRECT's artifact-aware normalization avoids the undesirable global data warping that happens when any damaged chips are processed without caCORRECT; (2) When used upstream of RMA, PLIER, or MAS5.0, the data imputation of caCORRECT generally improves the accuracy of microarray gene expression in the presence of artifacts more than using Harshlighting or not using any quality control; (3) Biomarkers selected from artifactual microarray data which have undergone the quality control procedures of caCORRECT are more likely to be reliable, as shown by both spike in and PCR validation experiments. Finally, we present a case study of the use of caCORRECT to reliably identify biomarkers for renal cell carcinoma, yielding two diagnostic biomarkers with potential clinical utility, PRKAB1 and NNMT.

**Conclusions:**

caCORRECT is shown to improve the accuracy of gene expression, and the reproducibility of experimental results in clinical application. This study suggests that caCORRECT will be useful to clean up possible artifacts in new as well as archived microarray data.

## Background

The reproducibility and reliability of microarray data is a major issue that must be addressed before microarrays can reach their full potential as a clinical molecular profiling tool for personalized and predictive medicine [1]. The FDA has completed phase-I of the MicroArray Quality Control (MAQC) project, which demonstrated general reproducibility among different array platforms and PCR, but came just short of offering concrete guidance on which processing methods to use when analyzing microarray data [2]. Recently published results from MAQC-phase II efforts demonstrate that well-designed microarray-based classification is reliable across experiments, and that in some cases, microarray-based classification can outperform existing clinical predictors [3,4]. The current status of microarray quality control (QC), however, is still a relatively anecdotal and inexact science based around a handful of existing methods. Tools such as dChip [5,6], MAS5.0 [7], RMAExpress [8-10], and PLIER [11] have been developed to improve the accuracy of microarray gene expression data by taking advantage of Affymetrix's high-density array design. These model-based tools use perfect match (PM) and mismatch (MM) information as well as the redundancy inherent in a probe set to generate estimates of gene expression, which are generally robust to failures of one or a few probes. While these tools use sensible methods of background correction, normalization, and statistical outlier detection, they fall short in two important areas. First, they do not incorporate adequate spatial information into the outlier detection methods and second, they do not incorporate outlier information into their normalization routines. caCORRECT [12] addresses these deficiencies and seeks to replace or augment existing methods to improve the reproducibility of microarray experimentation.

Quality Assurance (QA) tools, such as SmudgeMiner [13] and arrayMagic [14] provide intuitive images of damaged arrays through the use of heat maps, but they do not provide correction methods for observed errors. In fact, RMA and dChip also readily provide similar visualizations of chip errors, but they do not use that visualized information during probe outlier detection. Harshlighting [15,16] is similar to caCORRECT in that it identifies an assortment of compact and widely scattered artifacts, by leveraging techniques from the field of image processing, such as sliding windows and background assessment. Harshlighting, however, defines a chip's "error image" as a simple residual (i.e. subtraction) from the median. Harshlighting therefore ignores the differing natural variance of probes and neglects to account for global chip-to-chip variation, which is usually correctable with a simple normalization step. The R implementation of Harshlighting does allow for the input of user-generated error images, but this procedure is relatively skill-intensive. As a known issue, the authors of Harshlighting point out the appearance of "ghosting" artifacts i.e. the false appearance of artifacts on clean chips as a result of comparison to a true artifact on another chip in the batch. Whereas Harshlighting attempts to correct for this phenomenon by using a median in its error heat map calculation (as opposed to the more outlier-sensitive measure, the arithmetic mean), caCORRECT avoids the ghosting problem by iteratively identifying artifacts and directly omitting them from calculations altogether.

The LPE and CPP adjustments [17] have also been suggested as a way to correct spatial flaws on microarrays. Artifact probes are identified by LPE and CPP similarly to caCORRECT, i.e. their $d_{klr}^{*}$ measure is analogous to caCORRECT's *z*_*j *_and both methods use neighboring information. caCORRECT, however, allows for iterative calculation of this score, and thus allows for the same probe location to be corrected on more than one chip in a batch, whereas the methods of Arteaga-Salas et al. do not [12].

Previously, we have shown that using caCORRECT as a preprocessing step increases the reproducibility of biomarker selection as measured by similarity of ranked gene lists during independent cross-validation from large microarray datasets [12]. We have also shown that the spatial locations of proposed biomarkers (differentially expressed genes) in published microarray studies often are correlated or anti-correlated with the location of chip artifacts identified using caCORRECT [18]. Finally, we have constructed caBIG grid services for much of the functionality of caCORRECT [19]. Since these initial publications, improvements have been made to the caCORRECT algorithms, which have allowed more conclusive validations. Specifically, we have implemented a new bad-data-replacement algorithm (previously only median replacement was possible), and we have made user-centered design changes to allow more seamless integration with existing gene expression calculation protocols. caCORRECT's website currently offers gene expression output from RMA, PLIER, and MAS5.0. For users who wish to use other methods, such as the popular tool dChip, they can run the tools directly, using the clean cel file output option provided by caCORRECT. These validation results, which include the discovery of two biomarkers for renal cell carcinoma subtyping, as well as the description of improved methods are the subject of this manuscript.

## Results

### Artifact removal and replacement

**Figure 1:**
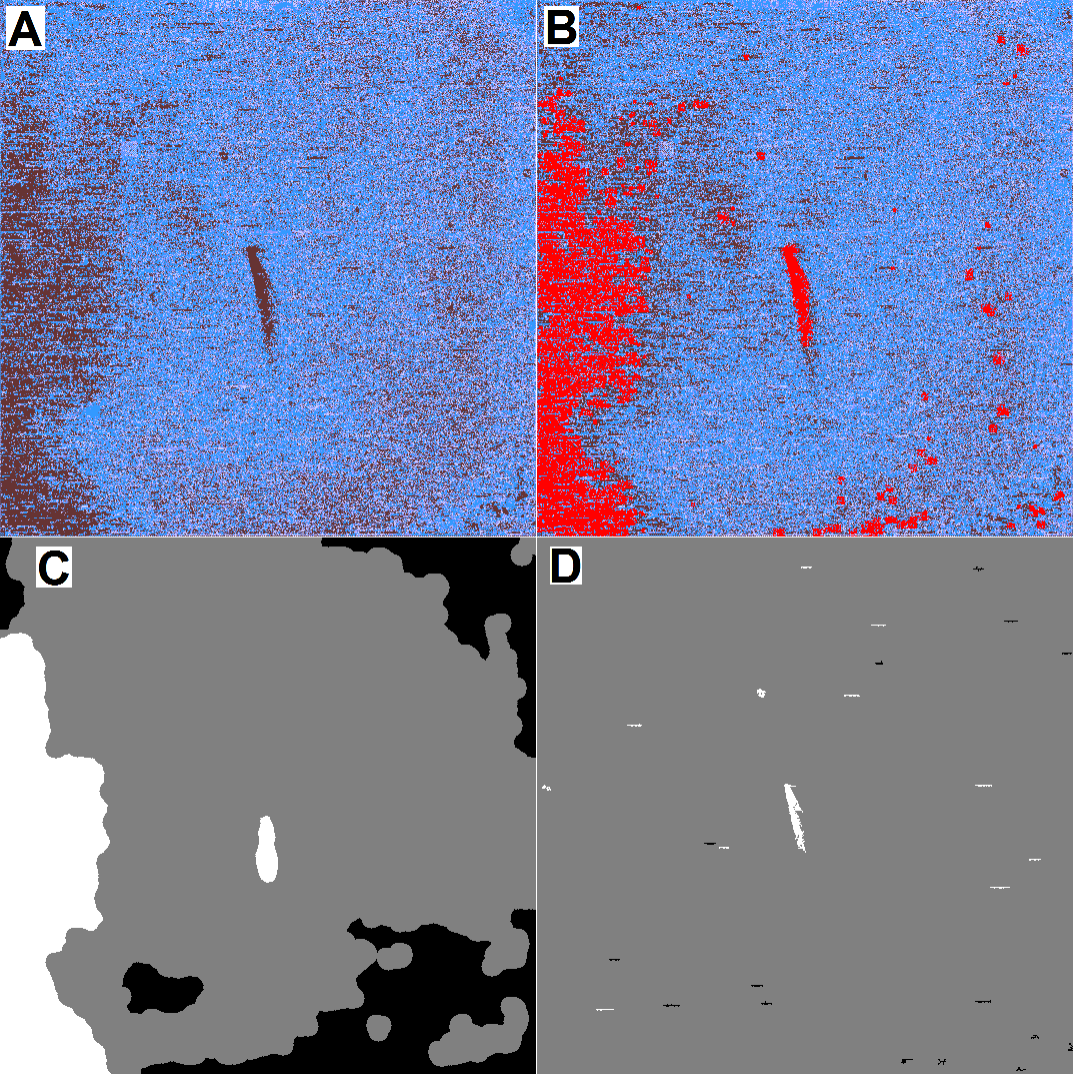
**Sample heat map and artifact segmentation result**. Panel A shows a heat map of the first variance score generated by caCORRECT for one chip in the West et al. dataset, with dark color indicating poor quality. Panel B shows the variance scores after artifacts have been flagged, with red indicating artifacts, and the rest of the probes colored by their final variance score as in panel A. Panel C and panel D show the compact and diffuse artifacts detected by Harshlighting in black and white with clean data in grey.

Many artifacts were easily visible using the heat map function provided by caCORRECT, and one such example of mixed artifacts observed in published data is shown in Figure 1. The input and output of caCORRECT's artifact identification procedure are visualized, demonstrating the process by which continuous scoring of the quality of individual probes (dark color indicating poor quality in panel A) is combined to determine the binary classification of "artifact or not" (artifacts shown in bright red in panel B). Note that not every probe with a poor score was flagged as an artifact, as local areas of high variance are expected as a result of actual variance in gene expression among samples. For comparison, the artifact labels provided by Harshlighting are shown for both diffuse and compact artifacts in panel C and D respectively, with black and white indicating artifacts. This example supports the general trend that caCORRECT's automated artifact identification is conservative. It is our experience that human observers often consider a larger portion of a chip more objectionable than caCORRECT does. Harshlighting on the other hand, tends to be more aggressive in flagging regions of chips as artifactual. We chose conservative artifact identification over a more aggressive scheme because caCORRECT was designed to identify spatial artifacts upstream of the more specific model-based outlier detection employed by most probe summarization methods, such as RMA, PLIER, and MAS5.0. Generally speaking, probe summarization is the fitting of a regression model to the probe intensity data from a chip or set of chips. While individual methods differ on how the model is set up (notably how they handle information from mismatch probes), they are the same in that the model-fitting procedures allow for different weights or affinities for each probe's contribution to gene expression. During this model-fitting process, single outlier probes are easily detected and ignored because they do not fit the model well. caCORRECT is not designed to detect or remove such isolated, single probe defects, because RMA, MAS5.0 and PLIER are already very good at this task. With large artifacts that affect many probes, however, model-based outlier removal becomes much more difficult. To help users interpret the success or failure of the probe summarization (i.e. model-fitting) process, client software such as RMAExpress, and now caCORRECT, visualizes the residuals (difference, or error) of every probe intensity from the model collectively as a heat map. Close interpretation of these residuals reveals how well a particular model fits the data.

**Figure 2:**
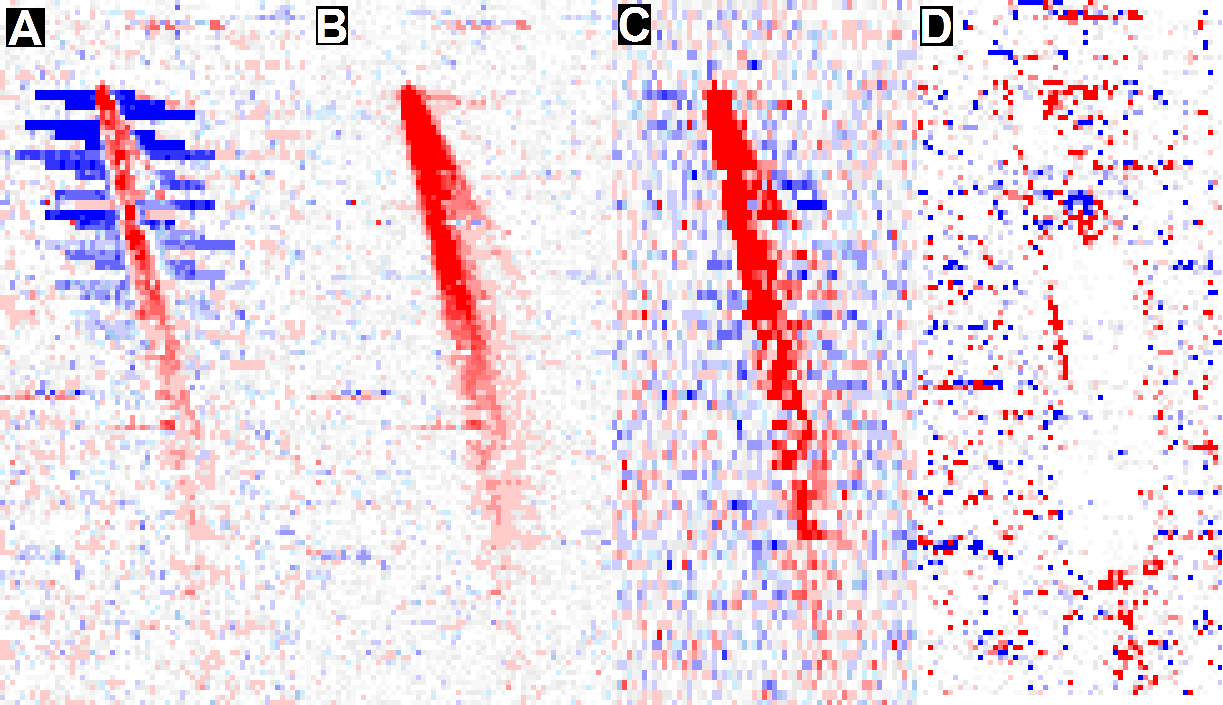
**Effect of scratch artifact and removal on residual images**. All 4 panels are heat maps with high positive values colored in red, values near zero in white, and large negative values colored in blue. The panels were not all generated identically, (i.e. panel C by RMAExpress, and others by caCORRECT), and so the scales and ranges may differ without loss of central meaning. All images show residuals between observed intensity and that expected by the underlying model. Thus, red color indicates higher than expected intensity, blue color indicates lower than expected intensity and white indicates good fit to the model. Panel A shows (caCORRECT) residuals from the original chip, panel B shows the (caCORRECT) residual after artifact flagging by caCORRECT, panel C shows the (RMA) residual produced by RMAExpress from the original chip, and panel D shows the (caCORRECT) residual produced after median-replacement form Harshlighting. Systematic blue color surrounding the red scratch (prominent in A and weaker in C) reveals poor model fit to the data, which is likely to cause an overestimate of gene expression for these probes. The figure suggests that caCORRECT has identified this scratch better than RMA has, and modeled the data better than Harshlighting has, resulting in a more accurate gene expression than either. The effective reduced image resolution for the RMA panel is due to the way that RMA handles pairs of perfect match (PM) and mismatch (MM) probes together when calculating residuals, whereas caCORRECT processes PM and MM probes independently of one another. Portions of this figure are reproduced with permission from [23].

Figure 2 represents a case study using residual images to compare how caCORRECT and RMA react to the presence of a scratch on an Affymetrix Hu-6800 chip from the West dataset. We chose a data set based on this older array platform, because, on the Hu-6800 chip, all probes which contribute to a single gene expression value are actually arranged in contiguous regions on the microarray (as opposed to newer chip layouts which distribute related probes randomly). This property allows for easier visual interpretation of residual images. Panel A of Figure 2 shows the residuals produced by the caCORRECT regressive gene expression model before any attempt to identify artifacts. The blue (negative residual) regions that surround the main red scratch (positive residual) demonstrate the ambiguity that arises when more than one outlier is present in a probe set. Without prior knowledge, the regressive model cannot determine whether the artifact data are too high, or the non-artifact data are too low. caCORRECT, however, identifies the actual scratched region as artifact, and the surrounding data as non-artifact. With this knowledge, the regressive model fits only to the non-artifact data. The resulting residuals that are calculated after caCORRECT are shown in panel B of Figure 2. The white region surrounding the scratch (and redness of the scratch itself) is evidence that caCORRECT has successfully disambiguated the decision. The data within the scratch are to be ignored, and the resulting model should use only the surrounding probe data to estimate gene expression. Panel C of Figure 2 shows residuals to the model produced by RMAExpress alone. RMA includes its own outlier detection, but RMA's detection is not informed by spatial location, and thus cannot recognize a scratch as such. The output from RMA suggests that RMA does a fair job of disambiguating the scratch from the surrounding data, but some areas of blue still exist near the scratch, suggesting that the gene expression values for these probe sets have been overestimated. Finally, the residual image produced after the median data replacement of Harshlighting, shown in panel D, suggests that most if not all of some probe set calculations are based solely on the median-replaced data, with non-artifact data in surrounding regions being regarded as outlier data. These problems have been almost completely avoided by caCORRECT, however.

### Effect of artifact aware quantile normalization on synthetic artifact data

**Figure 3:**
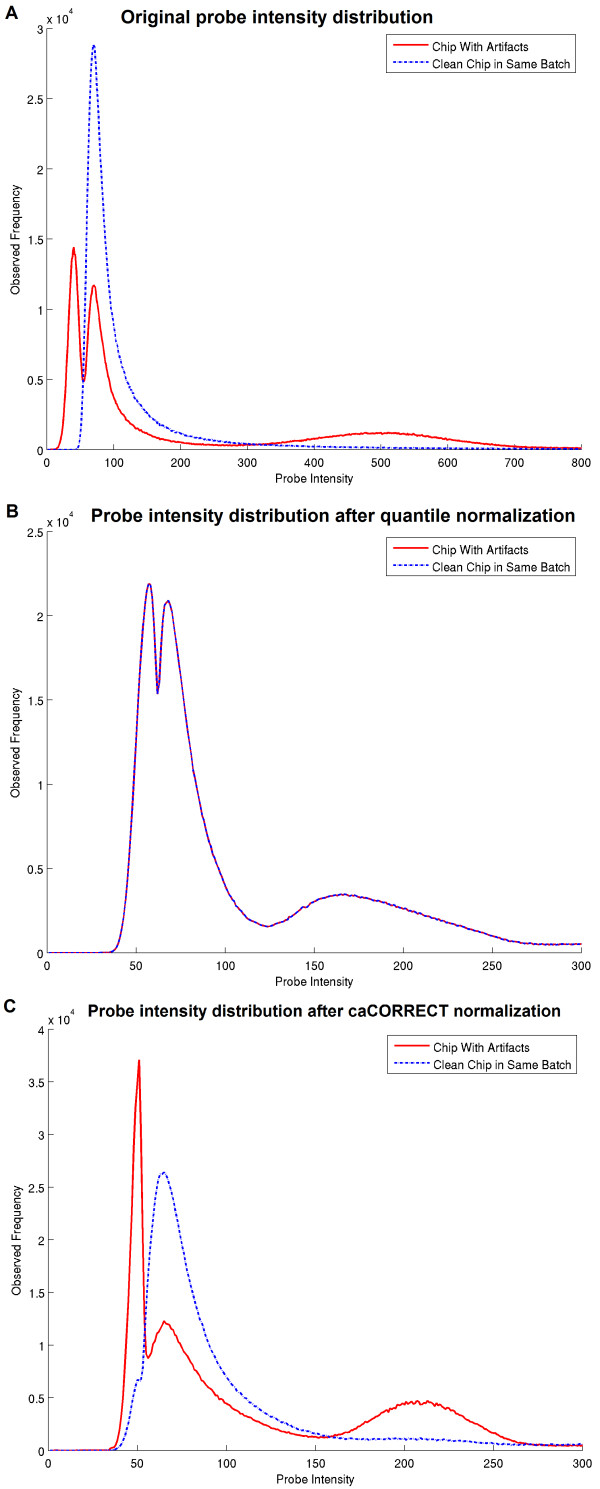
**Effect of quantile normalization or caCORRECT artifact-aware normalization on probe intensity distribution**. The top panel shows the distribution of probe intensities of two microarray chips, one of which has been affected by both a high intensity and a low intensity artifact (red), and the other which is of good quality (blue). The middle panel shows the distribution of these chips after quantile normalization. The bottom panel shows the distributions of these chips after caCORRECT artifact-aware quantile normalization.

Figure 3 illustrates the difference between standard quantile normalization and artifact-aware normalization. In this experiment the Schuetz et al. dataset has been modified with two large artifacts on one chip for illustrative purposes. As can be seen in the raw intensities (top panel), the synthetic artifacts (leftmost and rightmost modes in the red curve) cause a differently shaped distribution than that of a high quality chip (blue curve). Once standard quantile normalization is performed, a 'warping' of the high quality chip can be seen in the way that each of the distributions is now identically and incorrectly trimodal (middle panel). After caCORRECT artifact-aware normalization has been performed, the high quality chip returns to its natural distribution (bottom panel, blue curve). The chip with the artifacts still has two clear modes for its artifacts, but now the remaining non-artifactual data on the chip with the artifacts (central mode, red curve) has been properly normalized and aligned to the data from the high-quality chip (blue curve).

### Effect of applied artifacts and preprocessing on gene expression

**Figure 4:**
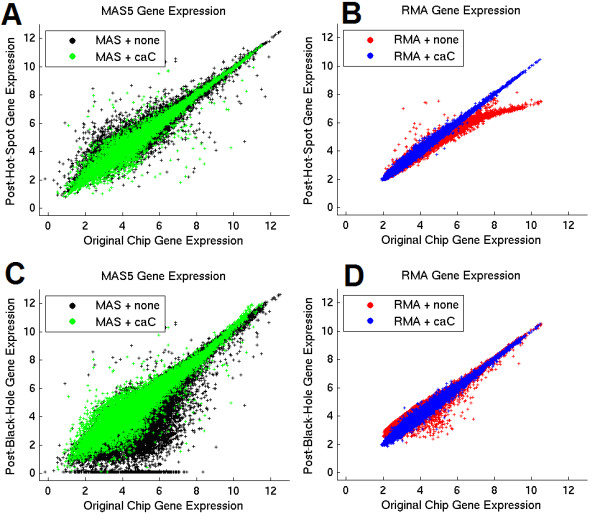
**Scatter plots of gene expression after quality insult versus original gene expression**. Data shown are for one representative chip, and all probe sets on the HG-U133A platform. Gene Expression is calculated either independently with MAS5.0 or with RMA as part of a batch containing the 81 independent chips in the original Hess et al. training set. caCORRECT normalization is performed independently for each chip as part of a batch with the 81 chips of the Hess et al. training set. Units of gene expression are on the scale of the natural log of probe intensity. caCORRECT improves gene estimation in all cases, as exhibited by scatter plots closer to a line with unitary slope. This figure has been reproduced with permission from [23].

We monitored the effect of applied quality insults on gene expression using the two popular probe summarization methods of RMA and MAS5.0. Figure 4 demonstrates the correspondence of gene expression, before and after introduction of artifacts by an independent third party. Two phenomena are readily observable:

First, for the "black hole" artifacts which lower probe intensities on the microarray, the MAS5.0 algorithm has the tendency to call many of the genes 'absent', and report the gene expression abnormally low (Figure 4 panel C). caCORRECT is able to almost completely reverse this trend, and is able to help MAS5.0 produce appropriate gene expression values for most of these probe sets.

Second, for the "hot spot" artifacts that raise probe intensities on the microarray, the RMA algorithm has the tendency to underestimate gene expression and lose accuracy for the genes most highly expressed in the sample (Figure 4 panel B, red). This is presumably a result of the warping issues related to quantile normalization discussed in the previous section. This phenomenon also happens to low-expressing genes in RMA to a lesser extent for the black hole artifacts (Figure 4 panel D, red). Chips processed first with caCORRECT and then with RMA do not exhibit either of these warping behaviors (Figure 4, blue).

We then created our own synthetic insults in order to compare caCORRECT with Harshlighting for the ability to moderate effects of single artifacts on gene expression, as measured by the probe summarization methods RMA, PLIER, and MAS5.0. As a control to these common methods, which include normalization and outlier detection inherently, we also measured expression with our simple *x*_*b,p,j *_= *θ*_*p*,*j*_*a*_*b,p *_+ *ε*_*b,p,j *_regression method "TAXY" detailed in the methods section, which does not contain special considerations for outlier data. Three conditions were tested for each method of gene expression measurement: (1) using original data that was not preprocessed; (2) using data that was processed with caCORRECT; and (3) using data that was processed with Harshlighting. We calculated the error caused by a given artifact as the average absolute difference, in the log domain, between the original gene expression and the expression measured after introduction of artifact. This measure is similar in concept to average relative error in gene expression in the linear domain.

**Figure 5:**
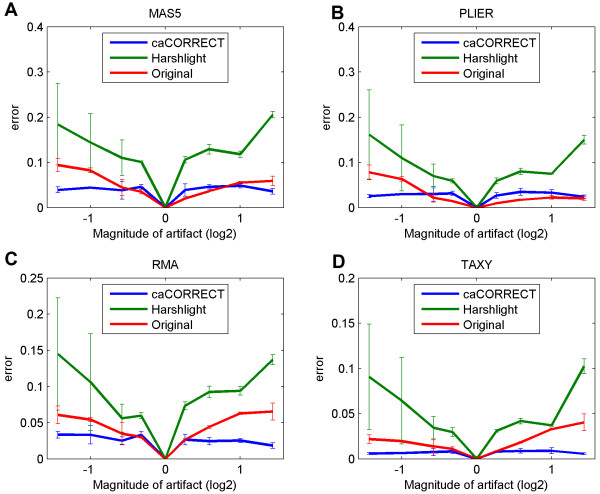
**Effect of artifact magnitude and preprocessing procedure on error of gene expression estimation**. The size of applied artifacts was fixed at 75 probes in diameter. Error is calculated as the average absolute difference, in the log domain, between original gene expression and expression measured after addition of artifacts. Error bars represent the standard deviation calculated across 3 trials.

Figure 5 shows the effect of artifact magnitude on gene expression. When the magnitude of the applied artifact was increased, the magnitude of error then increased for data that was not preprocessed, as well as for data processed with Harshlighting. In contrast, the magnitude of observed error remained constant, or even decreased, for data processed with caCORRECT. We expect that this is due to the phenomenon that as artifacts become more severe, they also become more obvious to caCORRECT. Even though harsher artifacts introduced more overall noise to the system, caCORRECT became better at identifying and removing them.

For RMA, caCORRECT outperformed both Harshlighting and unprocessed data for artifacts stronger than 1.5 fold. For PLIER and MAS5, however, caCORRECT outperformed unprocessed data only for intensity reducing artifacts. This suggests that caCORRECT is not suitable for helping MAS5.0 or PLIER to identify subtle artifacts, but that in the case of such subtle artifacts, caCORRECT does not reduce performance.

**Figure 6:**
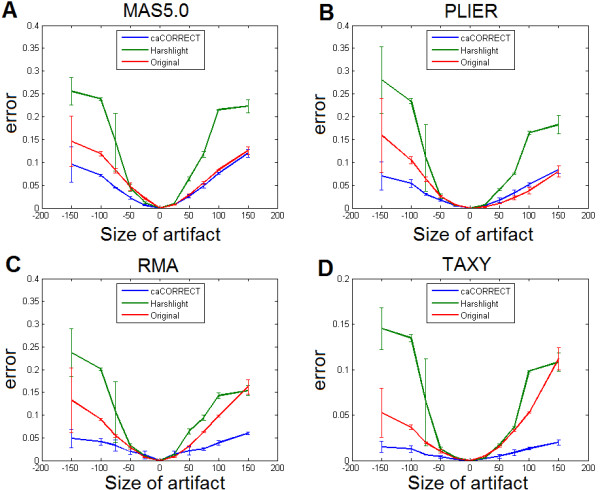
**Effect of artifact size and preprocessing procedure on error of gene expression estimation**. The size of the artifact describes the diameter of the circular artifact, with negative sizes indicating magnitude-reducing artifacts. The magnitude of applied artifacts was fixed at 2 fold (increase) or 0.5 fold (decrease) probe intensity in the footprint of the artifact. Error is calculated as the average absolute difference, in the log domain, between original gene expression and expression measured after application of artifacts. Error bars represent the standard deviation calculated across 3 trials.

Figure 6 shows the effect of artifact size on gene expression. With the magnitude of artifact fixed, an increasing artifact size increased error for each gene expression and preprocessing method tested. For RMA, caCORRECT outperformed both Harshlighting and unprocessed data for artifacts larger than 25 probes across. For PLIER and MAS5, however, caCORRECT outperformed unprocessed data only for intensity-reducing artifacts. Overall, results suggest that the size, magnitude, and sign of an artifact all affect the final error in gene expression differently, depending critically on the gene expression method being used.

### Identification of differentially expressed genes

**Figure 7:**
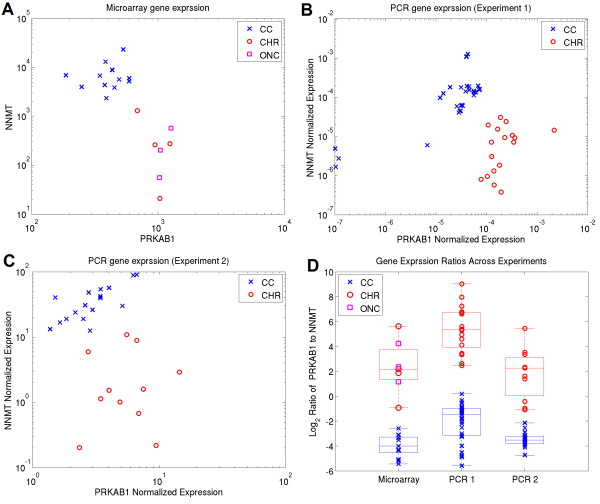
**Gene Expression of NNMT and PRKAB1 Biomarkers across Multiple Tissues and Platforms**. The differences in gene expression among three common subtypes of renal cell carcinoma are shown. In all panels, blue Xs and "CC" denotes clear cell, red Os and "CHR" denote chromophobe, and magenta squares and "ONC" denote Oncocytoma. (Panel A) Gene expression output from microarray data using MAS5, (Panel B) Gene expression from the first PCR experiment performed by the authors, (Panel C) Gene expression from follow-up PCR study #2 performed at a core facility, (Panel D) Comparison of expression ratios across tissues and platforms. Box and whiskers plots show the median, q1, q3, max and min values for samples grouped by subtype.

Having established that caCORRECT could improve the accuracy of gene expression derived from microarrays in the presence of spatial artifacts, we set out to determine if this improved accuracy of gene expression would translate to improved efficiency in identifying candidate genes to serve as biomarkers for disease. Briefly, we first identified differentially expressed genes between subtypes of renal cell carcinoma from the Schuetz et al. microarray data, then we performed a small PCR pilot study to verify these findings, and finally we tested a much larger cohort of genes and samples via a PCR core facility. While results could not be directly quantitatively compared between experiments, trends in gene expression, such as ratios among biomarkers were generally preserved. Figure 7 shows the trends in gene expression for the two most reliable biomarkers identified during this process: NNMT and PRKAB1.

**Figure 8:**
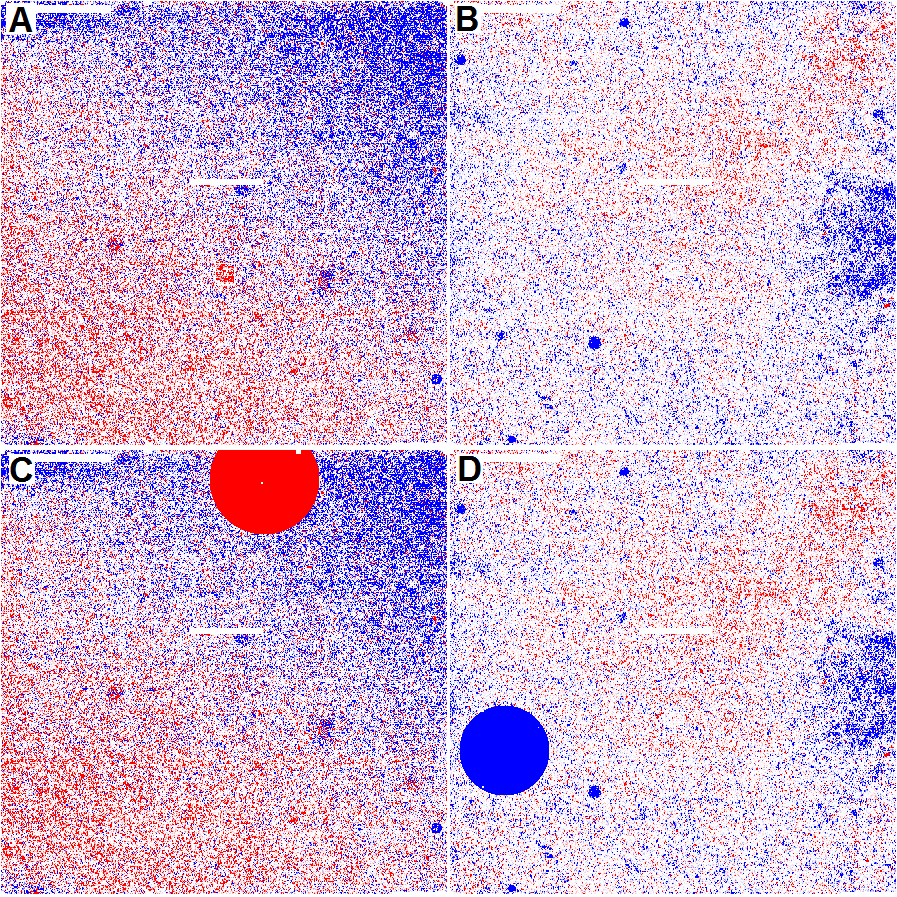
**Examples of artifacts present for biomarker identification analysis**. Images are heat maps of residuals for two pairs of arrays, with red color indicating higher-than-expected probe intensity, and blue color indicating lower-than-expected probe intensity. Heat maps such as these reveal the intensity and type of artifacts after caCORRECT normalization and artifact identification, but before removal. Panels A and B show naturally occurring artifacts on two chips from the Schuetz et al. dataset. Blue color in the upper right quadrant of panel A indicates data in this region is artificially low. Multiple small compact artifacts, both red and blue, can also be seen. Panel B shows more examples of naturally occurring artifacts, including a large artifact on the right edge of the chip. In panels C and D respectively, synthetic circle-shaped hot-spot and black-hole type artifacts have been applied to the same chips from panels A and B. The artifact in C appears red due to its artificially high probe intensity values. In panel D, the synthetic black-hole artifact is visible as a blue circle.

During the first PCR pilot study, we found only anecdotal evidence suggesting that using caCORRECT on real data could improve the reliability of biomarker selection. We believed that this could probably be attributed to the relatively high quality of the original chips in the Schuetz et al. study, and thus decided to artificially reduce the quality of the data in order to amplify any affect that caCORRECT may have. Figure 8 shows examples of our synthetic artifacts (Panels C and D) applied to the Schuetz et al. dataset as well as the unaltered versions of those chips (Panel A and B) as visualized by the post-caCORRECT residual images. While the synthetic artifacts may appear more visually stunning than the artifacts "naturally" found in this dataset, they are comparable with those found on other microarrays, such as the one shown covering the left hand side of the chip in Figure 1.

**Figure 9:**
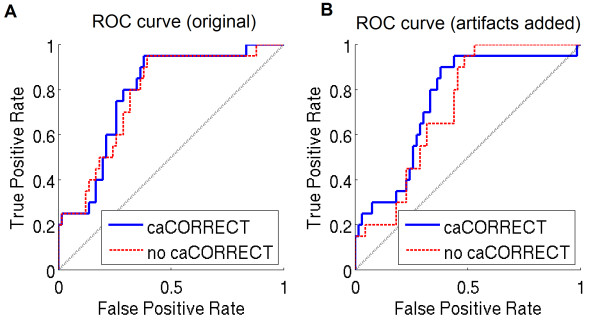
**Microarray Fold Change as a Predictor of PCR Fold Change in RCC Samples, and the Effect of Artifacts and caCORRECT Preprocessing**. Genes were thresholded by magnitude of observed log fold change in RMA-derived microarray data, and considered truly differentially expressed if they exhibited more than a 2x or less than a 1/2x fold change between classes CC and CHR in the PCR data. Only genes for which PCR data were available appear in this analysis. caCORRECT preserves data quality for arrays without serious artifact (area under the curve increase from 0.777 to 0.786) and improves quality for arrays that have serious artifacts (area under the curve increase from 0.723 to 0.751, but not all the way back to 0.777).

Figure 9 shows Receiver Operating Characteristic (ROC) curve analysis of microarray prediction (test prediction) versus PCR validation, which was defined as the gold standard for this purpose. For all cases, microarray data were generally predictive of follow-up PCR status. Results show that (1) caCORRECT was able to moderately improve biomarker selection power for typical microarray data (area under the curve increase from 0.777 to 0.786); and (2) caCORRECT was able to improve biomarker selection power for microarrays influenced by serious artifacts (area under the curve increase from 0.723 to 0.751). Importantly, caCORRECT had no undesired effects on the predictive power of clean data. Thus, caCORRECT should be suitable for datasets of unknown quality; in this setting, reliability would be expected to improve if the arrays contain significant artifact, while it would be unlikely to degrade if array artifacts are insignificant.

### Affycomp

**Figure 10:**
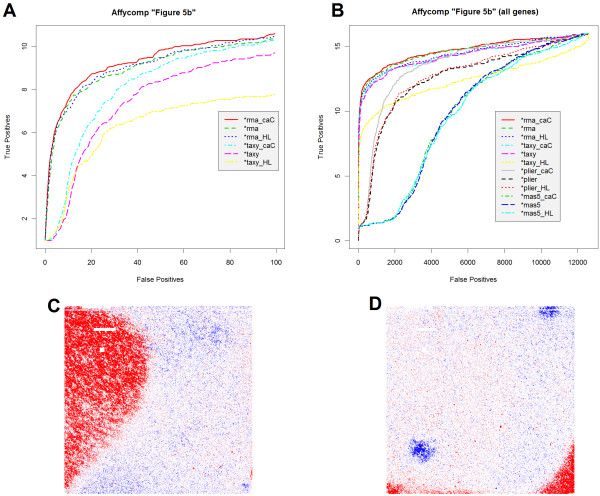
**Figures of merit derived from affycomp**. Panels A and B show figure of merit "5b" generated by affycomp for the HG-U95A spike in experiment, i.e. ROC curves for the task of identifying spiked in probe sets with known fold change of 2. Panel A is limited to 100 false positives, as suggested by Cope et al., while Panel B shows the same, but for all genes in the data set. Legend entries with caC denote that caCORRECT has been run on the data, and HL denotes that Harshlighting has been run on the data. "taxy" refers to the caCORRECT model of gene expression, while "rma", "mas5" and "plier" are computed with their respective R implementations. Panel C shows residuals (as colored in figure 8 and figure 2) for "chip 54" from the experiment, which has been hard-coded to be excluded from the analyses by the authors of affycomp. Panel D shows residuals for "chip21." caCORRECT improves performance for RMA, TAXY, and PLIER, but is unable to help the poorly-performing MAS5.0 for these data.

Cope et al. have provided a benchmark [20] by which to assess gene expression from two spike in datasets, one on the HG-U133A and the other on the HG-U95A platform [8]. Their spike in arrays were all prepared using the same cRNA stock mixture, with the exception of 16 transcripts which were added in concentrations from 0 to 1024 picoMolar. These 16 transcripts were applied (in triplicate) in a cyclic Latin square design. As a result, the fold change between any two arrays for these 16 transcripts is known, while the fold change for all other transcripts is expected to be 1 (no change). The affycomp package, available from http://bioconductor.org/, provides many statistics and figures of merit with which to compare the accuracy of gene expression in the context of detecting known fold change between pairs of arrays. Affycomp provides a uniquely public way to assess caCORRECT without the need to synthetically worsen the data. Figure 10, panels A and B, show caCORRECT's performance on the HG-U95A data set as a plot of false positive rate versus true positive rate (ROC curve). RMA after caCORRECT was the best overall performer, as measured by Area Under the Curve (AUC) for less than 100 false positives (0.830 for RMA after caCORRECT, 0.821 for RMA alone, and 0.818 for RMA after Harshlighting). AUC gains from caCORRECT were most noticeable for the TAXY method, which does not provide any additional outlier detection like that inherent in RMA, MAS5.0 and PLIER (0.760 for TAXY after caCORRECT, 0.740 for TAXY alone, and 0.719 for TAXY after Harshlighting). Importantly, the poorest-quality chip (Figure 10, panel C) was thrown out by the original authors as part of the canonical affycomp benchmark-- a decision which was recently justified quantitatively [21]. Still, examples of chips on which caCORRECT has detected obvious artifacts (Figure 10, panel D) remain in the HG-U95 spike in data. For the HG-U133A spike in dataset, which had less detected artifacts, improvements due to caCORRECT were more modest

It is almost never a good idea to run caCORRECT on batches with 3 or fewer chips, although it is hard to imagine a reliable microarray experiment so small. Affycomp data, however, followed a rarely achieved design in which there are 3 technical replicates of each sample. Arteaga-Salas et al. were able to apply their method for correcting small batches of replicates by splitting up the affycomp data set [17]. They have previously reported performance as the fraction of spike in genes from affycomp HG-U133A to have their RMA-calculated fold-change rank improved or worsened as a result of correction. Processing batches of 3 technical replicates at a time, they report 45.70% improved ranks, while caCORRECT, processing the entire dataset at once, improved ranks of 10.81% of spiked in genes. However, they report 38.60% worsened ranks, while caCORRECT worsened the ranks of only 9.52% spiked in genes. This result is consistent with caCORRECT's conservative design, but suggests that Arteaga-Salas's correction method may also be appropriate for experimental designs with available technical replicates for every sample.

## Discussion

caCORRECT is designed to correct spatial artifacts from batches of Affymetrix microarrays and to provide a robust global normalization before gene expression is calculated from the multiple probe values. Other sources of microarray noise which lack a spatial basis, such as RNA degradation, are not expected to be detected or altered by caCORRECT. Because modern chip layouts have more or less random arrangement of probes, these outlier probes are unlikely to be arranged in clusters on the chip surface large enough to be counted as artifacts. This same property also protects natural biological up regulation or down regulation of genes from being marked as artifacts. caCORRECT's performance is tied to both the size and quality of the batch being considered. First, the resolving power of artifact identification increases as the natural variance between samples decreases. Thus, the more similar samples are in a batch, the more powerful caCORRECT is. While technical or biological replicates are ideal, almost any cohort of arrays from the same study is an acceptable input. It is even possible to use caCORRECT to combine chips from two or more studies as long as they are from the same platform. Combination of data from different labs can easily introduce batch effects, however, and so this is generally not recommended. Second, even though sample size is accounted for within caCORRECT's variance score, the resolving power of artifact detection is diminished with smaller batches. For any size batch, but especially for those with less than 6 chips, we suggest watchful use of caCORRECT. Users should inspect the images provided by caCORRECT to confirm the quality of the data set. For chips with excessive artifact coverage (>50%), we suggest removing them altogether to avoid relying too much on imputation. We realize that this is not an attractive option for many researchers with small experiments, in which case we recommend including more chips or using caCORRECT only for quality assessment purposes.

While most existing gene expression algorithms include measures to remove artifacts, they are sub-optimal in that they ignore information about the spatial configuration of artifact probes. Using a visible scratch as an example, we have shown that caCORRECT's heat map-based outlier detection performs better than those methods that are purely based on statistical analysis of spatially-independent probe data. Blemishes such as the ones shown in this case study are common in microarray data and should be ignored or down-weighted when calculating gene expression.

Quantile normalization has been widely adopted by the microarray community as a way to remove global chip bias. We have shown that quantile normalization, while generally useful, can be counter-productive in datasets that have a chip with significant artifacts. First, good data from a chip with artifacts will be wrongfully displaced during normalization, i.e. high intensity artifacts will lead to underestimation for probe sets not in the footprint of the artifact. Second, probe data from otherwise clean chips or clean regions on damaged chips may be corrupted or distorted during quantile normalization if artifact data appear anywhere in the batch. caCORRECT alleviates this corruption by employing an artifact-aware quantile normalization scheme that is less susceptible to such data corruption or warping.

As an extension of the pitfalls of both the normalization and artifact identification schemes provided by modern microarray processing software, we show that caCORRECT combines advances in these two areas to improve overall accuracy of gene expression calculation. When operating upstream of PLIER, MAS5.0, and RMA algorithms, caCORRECT reduces the error in gene estimation, especially for cases of expression-lowering artifacts in MAS5.0, and expression-raising artifacts in RMA. The former effect is most likely influenced by MAS5.0's tendency to declare transcripts as *not present*, while the latter trend is most likely due to RMA's use of quantile normalization.

In contrast to caCORRECT, Harshlighting was observed to increase error in gene expression in most cases. Although the artifact segmentation results of Harshlighting are visually intuitive, the median based data replacement scheme appears to be unhelpful when used upstream of smart gene expression software. This is probably due to the fact that the median is a poorer estimate of the expected probe intensity than the replacement from model-based methods used by caCORRECT and probe summarization software. This is consistent with Troyanskaya et al.'s findings that singular value decomposition imputation outperforms mean replacement in the context of replacing missing gene expression values [22]. It appears that for most artifacts, the median replacement may imply false confidence, while a more extreme outlier, if left alone, may be detected and corrected by the simple methods inherent in RMA, PLIER, dChip, or MAS5.0.

Although we have shown that using caCORRECT improves the accuracy of derived gene expression data and the assessment of fold-change between pairs of arrays, this improvement in gene expression data quality has yielded only modest improvement in the reliability of biomarkers identified from a cohort of RCC samples. Specifically, we have shown using ROC area under the curve analysis that caCORRECT can improve the reliability of biomarkers identified from data affected by severe chip artifacts, without degrading performance on clean data. For the task of identifying differentially expressed genes from a cohort, much redundancy exists in the data themselves, and the impact of a single bad quality chip on the overall experiment is understandably small. The largest impact of caCORRECT is expected in applications which are relatively data-poor, or where the information on a single array is precious. The affycomp benchmark is such a "data-poor" application where differential expression is assessed based on head-to-head comparisons. The evidence that caCORRECT improves fold change assessment in the affycomp data thus supports the hypothesis that caCORRECT may be more noticeable in data-poor situations. An application of this is a clinical scenario in which a cohort of arrays is used to train a predictive model, but a single microarray is used to determine diagnosis or treatment decisions for a single patient. While throwing out a poor quality array may be suitable practice during model training, it is not an option during testing. A method such as caCORRECT could prove to be the difference between a correct and an incorrect clinical decision.

## Conclusions

We have demonstrated two fundamental reasons why caCORRECT represents a theoretical improvement over previous methods, as well as empirical evidence showing improved performance in gene expression accuracy and subsequent biomarker selection in the presence of severe artifacts and in the affycomp data. We expect that caCORRECT will be helpful for new experimentation as well as for revisiting the conclusions of archived microarray data that may suffer from artifacts.

## Methods

For all "caCORRECT" in this manuscript, version 2.1 of caCORRECT was used, as provided at http://cacorrect.bme.gatech.edu. The canonical bioconductor R-implementations of Harshlighting, RMA, MAS5.0, PLIER, and affycomp were also used. What follows in this section is a brief review of the previously published caCORRECT procedures which are pertinent to this study, as well as a description of the improvements which have been made since previous publication [12]. Much of the following text has been reproduced with modification from RAM's doctoral dissertation [23].

### Variance scoring

The cornerstone of caCORRECT's outlier detection is the concept of variance scoring, which is a description of each probe's tendency to be an outlier. Calculation of this score, *h*, is similar to conducting a t-test for whether or not the observed probe intensity for a given chip belongs to the observed distribution of probe intensities for all *other *chips in the dataset. A key feature of caCORRECT is that this distribution is estimated and updated multiple times during the course of a single caCORRECT session. Because of this dynamic updating, it is possible to identify subtle artifacts or pardon false artifacts that may have been misdiagnosed initially. Please refer to File01_supplement.pdf, "Supplemental Methods", for a more detailed description of how the variance score is calculated.

### Artifact segmentation

Once the variance statistic, *h*, has been calculated for each probe on each chip, false-color heat maps of *h*, showing probes in their original spatial layout, are generated to display regions of high noise. For a good quality microarray chip, *h *will represent biological variation in RNA expression for the sample. In this case, *h *will be distributed independently and nearly-uniform in magnitude throughout the chip. More commonly, however, protocols do not achieve uniform hybridization due to uneven drying, formation of salt streaks, scratching of the microarray surface due to contact with skin or dust, miscalculated hybridization times, or failure to control environmental variables such as ozone [24]. All of these most common mistakes result in localized regions of large *h *(artifacts) on the heat map.

caCORRECT uses a simple sliding window method to flag probes that meet two conditions: (1) they exist in regions of other high *h*-scoring probes, and (2) they have high *h *scores themselves. These two conditions ensure that most of the obvious artifacts are caught, but that most of the naturally occurring biological variance goes unnoticed. Because the intended platform for caCORRECT is a web-based grid service, artifact identification has been streamlined for speed and memory efficiency. More computationally intense methods such as active contours, PDE-based methods, or shape matching have been excluded in favor of a quick marching window algorithm that seems to work well for a wide range of data. To remove any global chip effects that arise from sample preparation or amplification, normalization is performed as described in the following section.

### Artifact-aware normalization

Quantile normalization reduces noise in microarray experiment replicates by forcing the intensity distribution of each chip to be identical [Bolstad B: Probe level quantile normalization of high density oligonucleotide array data, 2001]. The critical assumption behind quantile normalization is that for large genome-wide studies such as microarrays, the number of genes that are invariant to the experimental variables far outnumbers the number of biomarkers-- genes that respond to or predict experimental variables. Quantile normalization is generally good for the microarray problem, where the distributions are poorly defined and parametric methods such as median centering or Z-score normalization have their underlying assumptions violated. The power of quantile normalization comes with a major caveat. If the probe intensities of the chips are not distributed similarly, the algorithm will indiscriminately warp all the distributions to be the same, including any that may have been correct initially. Fortunately, it is a reasonable assumption that high-quality microarray data from a single source on a single platform follow the same distribution. Unfortunately, this high quality assumption is not valid for much real-world data, where chip artifacts can significantly alter the distribution of intensities on a chip. One bad chip can warp the others when quantile normalization is performed, thus compromising the reproducibility of the entire data set. A way to alleviate this problem is to identify artifacts before quantile normalization, and set them aside temporarily. In theory, perfect knowledge of artifacts would allow for perfect correction. This process is called "artifact-aware quantile normalization." caCORRECT uses four iterations of artifact-aware normalization and artifact identification in order to achieve a near steady-state normalization result with a relatively small amount of computation time.

To illustrate the invasive effect that artifacts can have on a dataset when quantile normalization is performed, synthetic microarray data were generated in the following manner: Six high-quality chips from the Schuetz et al. dataset [25] were chosen, one of which was set aside to receive artifacts. One third of the selected chip was modified by a multiplicative factor of 0.5, representing a low-intensity artifact. A different third of the selected chip was modified by a multiplicative factor of 10, representing a high-intensity artifact. These six chips were then processed using caCORRECT, and the probe intensities were monitored for warping at each intermediate step.

### Artifact replacement and probe intensity model

Once artifacts have been identified, a *clean *dataset is generated with the artifactual data appropriately replaced. The current version of caCORRECT uses a data imputation that is mathematically similar to the model used by RMA and others [5,9,10]. Notably, so-called perfect-match and mismatch probes are treated identically, i.e. we do not use PM-MM. In this scheme, the artifact-flagged probe level data is replaced with the best-fit estimate for that probe, given the model and data from non-artifactual probes on all of the chips being processed.

Observed microarray intensity values after global normalization are modeled as a multiplicative combination of target RNA abundance (gene expression) and probe-specific effect (probe affinity). The model is given as:

$$x_{b,p,j}=\theta_{p,j}a_{b,p}+\varepsilon_{b,p,j},$$

where *x*_*b,p,j *_is the observed intensity for the *b*^*th *^probe in the *p*^*th *^probe set on the *j*^*th *^chip, *θ*_*p,j *_is the gene expression term, *a*_*b,p *_is the probe affinity term, and *ε*_*b,p,j *_is the additive error term.

The set of equations for the *p*^th ^probe set using an additive model of error can be represented in the following matrix form, given a set of *N *chips and *B*_*p *_probes in the *p*^th ^probe set.

$$X_{p}=\theta_{p}a_{p}+\varepsilon_{p},$$

where

$$X_{p}=\left[\begin{array}{ccc} x_{1,p,1} & \cdots \; & x_{B_{p},p,1}\\ \vdots & \ddots & \vdots \\ x_{1,p,N} & \cdots \; & x_{B_{p},p,N} \end{array}\right],$$

$$\theta_{p}=\left[\begin{array}{c} \theta_{p,1}\\ \vdots \\ \theta_{p,N} \end{array}\right],$$

$$a_{p}=\left[\begin{array}{ccc} a_{1,p} & \cdots \; & a_{B_{p},p} \end{array}\right],$$

and

$$\varepsilon_{p}=\left[\begin{array}{ccc} \varepsilon_{1,p,1} & \cdots \; & \varepsilon_{B_{p},p,1}\\ \vdots & \ddots & \vdots \\ \varepsilon_{1,p,N} & \cdots \; & \varepsilon_{B_{p},p,N} \end{array}\right].$$

We define the solution to this matrix equation as that which minimizes the Frobenius norm of the above error matrix *ε*_*p *_as defined below.

$${∥\varepsilon_{p}∥}_{F}=\sqrt{\overset{N}{\underset{j=1}{\sum }}\overset{B_{p}}{\underset{b=1}{\sum }}{\left(\varepsilon_{b,p,j}\right)}^{2}}$$

The solution which satisfies the above condition can be derived from the singular value decomposition (SVD) of X_*p*_. Here, the SVD of X_*p *_is given in the form of X_*p *_= USV^T^, such that,$U\in {ℜ}^{N\times N}$, $S\in {ℜ}^{N\times B_{\text{p}}}$ and $V\in {ℜ}^{B_{\text{p}}\times B_{\text{p}}}$. If the largest singular value in **S**, *s*_1_, is arranged as the first diagonal element of **S**, then **θ**_*p *_is *s*_1 _times the first column of **U **and $a_{p}^{\text{T}}$ is the first column of **V**.

We chose to introduce the additional constraint that the geometric mean of the lumped probe affinity terms *a*_*b,p *_equals one. The number one is arbitrary here, but it allows the convenient interpretation that the values of gene expression, *θ *_*p,j*_, are on the same scale as the probe intensities, *x*_*b,p,j*_. To satisfy this constraint, the earlier solution for **a**_*p *_and **θ**_*p *_can simply be scaled by respective multiplication and division.

### Imputation of artifact values

With values of **θ**_*p *_and **a**_*p *_learned from SVD, the probe intensities which are expected from the model can be generated via the multiplication **θ**_*p*_**a**_*p *_Incorporation of artifacts into this model is done in the following 2-step Expectation-Maximization algorithm until **θ**_*p *_and **a**_*p *_converge.

1) Estimate model parameters **θ**_*p *_and **a**_*p *_from the SVD of the observed data **X**_*p*_.

2) Replace known artifact values in **X**_*p *_with information from the corresponding elements of **θ**_*p *_**a**_*p*_.

This procedure of replacing values in **X**_*p *_with values from **θ**_*p *_**a**_*p *_has the effect of reducing corresponding values of ε_*p *_to zero, and thus has the property of never increasing the Frobenius norm of ε_*p*_. Since step 1 is a global minimization of the Frobenius norm of ε_*p *_given X_*p*_, and step 2 alters X_*p *_in a way that can only further decrease this error, the entire procedure is guaranteed to converge due to the non-increasing nature of the error function, which is naturally bounded by zero. Troyanskaya et al. have previously used a similar "SVDImpute" procedure for missing gene expression data [22]. Please see additional file 1, "Supplemental Methods", for further details.

### Datasets and synthetic artifact generation

In order to quantify the ability of caCORRECT to improve data quality, we altered public microarray datasets with a variety of randomized synthetic artifacts, and then processed the altered data with caCORRECT or Harshlighting. Datasets were generated from a variety of clinical cancer studies using different microarray platforms. To date, the caCORRECT website has been tested with data from 18 different Affymetrix platforms, but the results of our synthetic artifact analysis which are presented here are limited to our in-depth study of two key data sets. Three separate experiments were performed involving application of synthetic artifacts.

### Third party artifacts on Hess et al. data

First, we obtained a large data set that was originally generated by Hess et al. [26] in the study of breast cancer, and then later used as part of MAQC phase-II study on classifier performance. This original data set consisted of 130 high-quality samples assayed on the Affymetrix HG-U133A platform. The Hess et al. study divided samples into training (n = 81) and validation (n = 49) sets and we retain this distinction. Chips in the validation set were selected for synthetic artifact manipulation by an independent team lead by Wendell Jones of Expression Analysis, (previously unaffiliated with caCORRECT). Two types of artifacts were investigated here: (1) a "black hole" artifact in which an elliptical region of the microarray had probe intensities lowered severely, and (2) a "hot spot" artifact in which an elliptical region of the microarray had probe intensities raised severely. Twenty digitally altered copies of each of the original 49 chips were prepared as follows: A single artifact with random orientation and location was applied to each chip (ten chips received "black holes", and ten received "hot spots"). For each of the altered chips, gene expressions were calculated both before and after caCORRECT's complete artifact detection and value imputation. Expression data for all probes were determined both using MAS5 in Expression Console and the R implementation of RMA. Each of the estimated gene expressions from the altered chips was then compared to the "true" gene expression values obtained from the respective original, unaltered chip to yield an error value representing the deleterious effect of the artifact on gene expression estimation. The errors for each probe set (22283), each chip (49), and each artifact replicate of a given type (10) were then pooled together to form distributions of errors of size n = 10,918,670. Eight such distributions were created in total, representing the combinations of two gene expression methods, two artifact types and either unprocessed data, or for data cleaned with caCORRECT.

### Artifacts generated on data from Hess et al

A common criticism of our synthetic artifact work is that the size and severity of tested artifacts (for example those provided by Jones' team) are rarely observed in practice. While we have encountered many chips plagued by large, severe artifacts, (see http://arraywiki.bme.gatech.edu/index.php/Hall_of_Fame for some examples [27]), we set out here to investigate how the magnitude and size of artifacts may affect our previous conclusions as to the usefulness of caCORRECT. Thus, we have created a second set of synthetic artifact data specifically for this purpose by our own application of artifacts to the same breast cancer dataset from Hess et al that Jones' team used. Only the 15 highest quality arrays (via visual inspection of heat map images) from this dataset were used in order to both speed up computation as well as to more precisely isolate the effects of our synthetic artifacts. For a variety of sizes and magnitudes, circular regions of the array were altered multiplicatively. Care was taken so that no more than 1/2 of the radius of the circular insult appeared off of the chip. The final gene expression obtained from the altered array was then compared to the gene expression obtained from the original unaltered array, and the average of the relative error for each probe set on the array was stored. For each pair of size and magnitude, this process was repeated a total of 3 times.

### Artifacts observed and generated on data from Schuetz et al

Finally, a realistic mixture of less-severe artifacts were applied to the Schuetz et al. [25] dataset in order to monitor the effect of typical artifacts on differential gene finding, and the ability of caCORRECT to ameliorate these effects. This data set consisted of 20 Renal Cell Carcinoma (RCC) samples assayed on the Affymetrix HG-Focus Platform by Schuetz et al. Samples were classified by tumor subtype: Clear Cell (CC), Oncocytoma (ONC), and Chromophobe (CHR). For biomarker selection purposes, samples were combined into two classes: seven CHR or ONC versus thirteen CC.

### Artifacts observed on data from West et al

The dataset which was used to showcase real-world artifact removal and replacement was a set of 49 Hu-6800 Affymetrix microarrays from the study by West et al. This study investigated Estrogen Receptor and lymph node metastatic status [28]. This data set is chosen because it used an older version of Affymetrix chip in which the properties of artifacts are more easily visualized than on modern chips.

### PCR Validation

To determine the effect that caCORRECT had on the ability to correctly identify biomarkers of disease from microarray data, a panel of 96 genes of interest for RCC was assembled for PCR study in two phases. These genes were identified from a combination of genes previously identified in the literature as well as a set of genes whose biomarker status was disagreed upon between the caCORRECT and non-caCORRECT versions of the Schuetz et al. data sets using omniBiomarker (http://omniBiomarker.bme.gatech.edu). All PCR analysis was performed on independent patient tissue samples with respect to those used for the microarray analysis.

Gene expression was assessed by quantitative RT-PCR, using total RNA from fixed tissues of 17 clear cell and 7 chromophobe RCC patients. Duplicate experiments were performed according to published protocols with minor modifications: Histological sections were deparaffinized with ethanol and xylene, and cells of interest were microdissected with a sterile scalpel. Tissues were digested in buffer containing proteinase K at 60°C overnight. RNA was extracted with phenol/chloroform, and genomic DNA was removed with DNase. RNA quality and quantity were assessed with a Bioanalyzer (Agilent Technologies). Up to 3 μg of RNA was used for first strand cDNA synthesis with Superscript III (Invitrogen). PCR was performed with a custom-designed Taqman Low Density Array (LDA, Applied Biosystems) in a 96-well microfluidic card format, using the ABI PRISM 7900HT Sequence Detection System (high-throughput real-time PCR system). Gene expression data were normalized relative to the geometric mean of two housekeeping genes (18S, ACTB). LDA runs were analyzed by using Relative Quantification (RQ) Manager (Applied Biosystems) software.

Relative normalized PCR gene expression was compared in renal tumor subtypes. Genes were declared as being "validated by PCR" if they had an average fold change between classes of magnitude greater than 2, corresponding to an average C_t _(threshold cycle) value difference of 1.

## Competing interests

The authors declare that they have no competing interests.

## Authors' contributions

RAM helped design caCORRECT, including artifact-aware normalization, variance scoring, and missing data imputation methods. RAM designed and performed all microarray-based validation experiments, helped perform PCR validation, and drafted the manuscript. QYG performed biological specimen preparation and PCR validation. THS helped design caCORRECT, including quality scoring, created the caCORRECT web portal and its companion ArrayWiki, and helped caCORRECT to receive silver-level compatibility certification from NIH/NCI caBIG. RMP helped design and prototype caCORRECT's data imputation. JHT participated in PCR experimentation and the collection of source microarray data. JHP implemented caCORRECT's normalization and performed all gene ranking done with SVM. ANY provided all biological specimens, contributed to manuscript preparation, and directed the PCR validation. MDW initiated the microarray quality control investigation, acquired funding to sponsor this multi-year effort, and directed the caCORRECT project, software system certification, and publication. All authors read and approved the final manuscript.

## Supplementary Material

Additional file 1 **Supplemental Materials**. Additional descriptions of methodology for: calculation of variance score, implementation of gene expression model imputation, biomarker selection, and PCR experimentation Click here for file
