# Supplementary material for: caCORRECT2: Improving the accuracy and reliability of microarray data in the presence of artifacts
Source: BMC Bioinformatics. 2011 Sep 29;12:383. doi: 10.1186/1471-2105-12-383 (PMC3230913; doi:10.1186/1471-2105-12-383)
Supplement: Additional file 1 — Supplemental Materials. Additional descriptions of methodology for: calculation of variance score, implementation of gene expression model imputation, biomarker selection, and PCR experimentation [file 1471-2105-12-383-S1.PDF]

Supplemental Methods

Much of the following text has been reproduced with few modifications from RAM's doctoral dissertation [1].

Calculation of Variance Score

To estimate the distribution of probe intensity for each probe on chip j, data from the other chips is used. First, an artifact adjusted mean μ_j is calculated by using the expression values x_i from other chips ($i \neq j$) in the dataset and artifact attenuation factor α_i. As a default, the attenuation factor equals one for spots that have not been identified as artifactual, and zero for spots that have been identified as artifactual. After this artifact adjusted mean is calculated, an artifact adjusted deviation score σ_j is calculated in a similar manner. These two statistics, μ_j and σ_j thus form a description of the null distribution against which x_j is to be compared. The final variance statistic z_j for a location is then calculated as the difference between the observed intensity x_j and the weighted mean μ_j, divided by the weighted standard deviation σ_j.

$$\alpha_i = \begin{cases} 0: & artifact \\ 1: & not\ artifact \end{cases} \qquad \mu_j = \frac{\sum_{i \neq j} x_i \cdot \alpha_i}{\sum_{i \neq j} \alpha_i}$$

$$\sigma_j = \sqrt{\frac{\sum_{i \neq j} \left[(x_i - \mu_j)^2 \cdot \alpha_i \right] \sum_{i \neq j} \alpha_i}{\left(\sum_{i \neq j} \alpha_i \right)^2 - \sum_{i \neq j} \alpha_i^2}} \qquad z_j = \frac{x_j - \mu_j}{\sigma_j}$$

The result of this calculation is a variance statistic, z, for each spot on each chip in the study. A high magnitude z indicates artifactual *tendency*, while a low magnitude z

suggests that the spot is to be trusted. Note that a high magnitude score could also be a result of biologically relevant gene expression, but those cases will be generally ignored during the artifact identification procedure described in the next section. A nonlinear scaling procedure is then applied to each z to yield h, which is a score between 0 and 1 that has been adjusted for the number of chips in the dataset, and is similar, but not equivalent to 1 minus a p-value. This adjustment is made to provide consistent visualization and image processing

$$h = 1 - \exp\left(-\left(\frac{z_j}{n} \right)^2 \right)$$

In the above formula, n is a scaling factor equal to the ratio of the p=0.05 critical point in the student's t distribution with degrees of freedom equal to one less than the number of chips used in the calculation of z_j, to the p=0.05 critical point in the Gaussian distribution. Since the number n increases with decreasing degrees of freedom, this scaling factor helps ensure that successive rounds of caCORRECT do not progressively and indefinitely shave off more and more outliers until there is nothing left. Importantly, h is set to zero if the number of non-artifact chips drops below 3. This statistic is used as a guideline for identifying regions of chips where artifacts are present.

Gene expression model implementation

If we assume that $\mathbf{X}_p$ is rank 1, (that it can be perfectly approximated by the multiplication of a single column vector $\boldsymbol{\theta}_p$ and single row vector $\mathbf{a}_p$), then we can use a shortcut to estimate $\boldsymbol{\theta}_p$ and $\mathbf{a}_p$ directly from $\mathbf{X}_p$ without using an SVD. In such a

situation the summation of the bth column of $\mathbf{X}_p$ would give a clue to the bth element of $\mathbf{a}_p$ as follows.

$$\sum_{j=1}^{N}\left(x_{b,p,j}\right)=\sum_{j=1}^{N}\left(\theta_{p,j}a_{b,p}\right)=a_{b,p}\sum_{j=1}^{N}\left(\theta_{p,j}\right)$$

This b^{th} column summation of $\mathbf{X}_p$ is defined as $\varphi_{b,p}$, which can then be combined into a row vector, $\boldsymbol{\varphi}_p$.

$$\boldsymbol{\varphi}_p=\begin{bmatrix}\varphi_{1,p} & \cdots & \varphi_{B_p,p}\end{bmatrix}=\begin{bmatrix}a_{1,p} & \cdots & a_{B_p,p}\end{bmatrix}\sum_{j=1}^{N}\left(\theta_{p,j}\right)$$

In this form, we can see that $\boldsymbol{\varphi}_p$ is simply a scaled version of $\mathbf{a}_p$. Because of the constraint on $\mathbf{a}_p$ that geometric mean be set to 1, $\mathbf{a}_p$ can be easily derived from $\boldsymbol{\varphi}_p$ by normalizing $\boldsymbol{\varphi}_p$ by its geometric mean as follows.

$$\mathbf{a}_p=\frac{\boldsymbol{\varphi}_p}{\sqrt[Bp]{\prod_{b=1}^{B_p}\varphi_{b,p}}}$$

Similarly, summation of the jth row of $\mathbf{X}_p$ would give a clue to $\theta_{p,j}$.

$$\sum_{b=1}^{B_p}\left(x_{b,p,j}\right)=\sum_{b=1}^{B_p}\left(\theta_{p,j}a_{b,p}\right)=\theta_{p,j}\sum_{b=1}^{B_p}\left(a_{b,p}\right)$$

Since the summation of $\mathbf{a}_p$ is calculable, so is each element of $\boldsymbol{\theta}_p$.

$$\theta_{p,j}=\frac{\sum_{b=1}^{B_p}\left(x_{b,p,j}\right)}{\sum_{b=1}^{B_p}\left(a_{b,p}\right)}$$

Biomarker Selection

All gene expression calculations for RCC Affymetrix chips were obtained using the RMA algorithm, using RMAExpress [2-4]. RMAExpress is a Windows GUI for the Robust Multichip Average (RMA) method commonly used through Bioconductor. RMAExpress was chosen here for its speed, lack of selectable options, and ability to easily produce residual images for each chip. When using RMAExpress, all data were processed using both the quantile normalization and background correction features included in the software.

To determine the ranked gene list for each dataset we used one-dimensional linear Support Vector Machines (SVM.) SVM was chosen because of its ability to generalize well, and its reliable error estimation, which make it ideal for selecting ranked lists of candidate biomarkers. The SVM was implemented in parallel on a 74-node cluster for speed and convenience, and is accessed through a web-based GUI, called omniBiomarker (http://omnibiomarker.bme.gatech.edu). The SVM classifier used a Gaussian bolstering kernel with a bolstering radius of 1.4826 and 100,000 points of Monte Carlo integration for error estimation. For more information on our implementation of SVM, its application to microarray study, and sensitivity to quality control, we refer you previous work [5-8]. The classification error output by the omniBiomarker SVM was then used as a basis to rank each gene.

Two separate ranked lists of biomarkers were produced from the RCC Microarray data before and after preprocessing with caCORRECT, referred to as pre-QC and post-QC lists respectively. Pre-QC and post-QC biomarker lists were then compared to select out subsets of genes fulfilling the following criteria:

Set 1: The gene must be inside the top 100 of the pre-QC list, and not in the top 100 of the post-QC list.

Set 2: The gene must be inside the top 100 of the post-QC list, and not in the top 100 of the pre-QC list.

Set 3: The gene must be inside the top 100 of both the pre-QC list, and the post-QC list.

The genes in each of these sets were then reduced, based on availability of suitable PCR primers.

Independent Biomarker Validation by PCR

Fifteen of the genes meeting these criteria were then quantified with PCR on a cohort of independent RCC samples. 8 samples each of Clear Cell and Chromophobe RCC were used for the pilot study, with cDNA extractions from adjacent slices of each FFPE tissue block constituting duplicate samples.

This PCR work was then expanded to a panel of 96 genes as described earlier in the main methods section. This panel was tested by the microarray core facility at Emory University using even more independent tissue samples. In the main manuscript this 96-gene panel is referred to as batch II, while the previous 15-gene panel is referred to as batch I.

References

1. Moffitt RA: **Quality control for translational biomedical informatics.** Georgia Institute of Technology, 2011.
2. Irizarry RA, Bolstad BM, Collin F, Cope LM, Hobbs B, Speed TP: **Summaries of affymetrix GeneChip probe level data.** *Nucleic Acids Research* 2003, **31:**-.
3. Bolstad BM, Irizarry RA, Astrand M, Speed TP: **A comparison of normalization methods for high density oligonucleotide array data based on variance and bias.** *Bioinformatics* 2003, **19:**185-193.
4. Irizarry RA, Hobbs B, Collin F, Beazer-Barclay YD, Antonellis KJ, Scherf U, Speed TP: **Exploration, normalization, and summaries of high density oligonucleotide array probe level data.** *Biostatistics* 2003, **4:**249-264.

5. Phan J, Moffitt R, Dale J, Petros J, Young A, Wang M: **Improvement of SVM Algorithm for Microarray Analysis Using Intelligent Parameter Selection.** *Engineering in Medicine and Biology Society, 2005 IEEE-EMBS 2005 27th Annual International Conference of the* 2005**:**4838-4841.
6. Moffitt R, Phan J, Hemby S, Wang M: **Effect of Outlier Removal on Gene Marker Selection Using Support Vector Machines.** *Engineering in Medicine and Biology Society, 2005 IEEE-EMBS 2005 27th Annual International Conference of the* 2005**:**917-920.
7. Sima C, Braga-Neto U, Dougherty ER: **Superior feature-set ranking for small samples using bolstered error estimation.** *Bioinformatics* 2005, **21:**1046-1054.
8. Cristianini N, Shawe-Taylor J: **An introduction to Support Vector Machines.** 2000.
